# Impressão de Protótipo Tridimensional (P3d) de Coração para Aplicação na Cardiologia Pediátrica: Uma Experiência Inicial

**DOI:** 10.36660/abc.20200086

**Published:** 2021-03-03

**Authors:** Maíra Levorato Basso, Alessandra Möbius Gebran, Julia Dullius Oliveira, Katrin Möbius Gebran, Letícia Carlota Bonatto, Maria Cecília Knoll Farah

**Affiliations:** 1 Hospital Pequeno Príncipe CuritibaPR Brasil Hospital Pequeno Príncipe, Curitiba, PR - Brasil; 2 Faculdades Pequeno Principe CuritibaPR Brasil Faculdades Pequeno Principe, Curitiba, PR – Brasil

**Keywords:** Cardiopatias Congênitas, Angiografia Coronária/métodos, Tomografia Computadorizada por Raios X/métodos, Impressão Tridimensional/tendências

## Introdução

A tecnologia de impressão de protótipos tridimensionais (P3D) desenvolve-se rapidamente na medicina. Estudos recentes mostram a importância de sua aplicabilidade na cardiologia pediátrica.

Profissionais, clínicos e cirurgiões fazem uso de tecnologias bidimensionais como a ecocardiografia, a angiotomografia computadorizada (ATC) e a ressonância magnética para caracterizar estruturas e entender a complexidade das doenças. De modo geral, essas imagens são projetadas e estendidas em tela plana, de modo a não representarem o tamanho real das estruturas, a percepção de profundidade ou a proximidade entre elas.^1^

As cardiopatias congênitas têm prevalência de 9,1/1.000 nascidos vivos^2^ e geram consequências hemodinâmicas e funcionais significativas, sendo responsáveis por 6% dos óbitos de crianças no primeiro ano de vida no Brasil.^3^ A diversidade e a complexidade das cardiopatias exigem do profissional a compreensão detalhada da anatomia no plano terapêutico, uma conduta adequada e a explicação didática aos familiares sobre a doença.^4^^,^^5^

Este estudo fez a impressão em P3D de cardiopatias congênitas a partir de imagens de arquivo de ATC e também como experiência inicial visando a construir uma maior evidência científica.

## Métodos

Trata-se de um estudo descritivo e observacional no qual não foi aplicado um instrumento de comparação numérica. No entanto, visa a ressaltar uma experiência inicial, considerando a evidência presente na literatura médica sobre o uso da tecnologia 3D e os benefícios na compreensão das patologias cardíacas congênitas e sua importância em nosso meio. Imagens representativas das cardiopatias foram obtidas de arquivos de ATC, escolhidas dentre as que apresentavam maior complexidade anatômica e de acordo com a obtenção de melhores informações estruturais com a reprodução tridimensional. As cardiopatias selecionadas foram: atresia pulmonar com comunicação interventricular (APu+CIV) e colaterais sistêmico pulmonares, e hipoplasia do coração esquerdo (SHCE) após a colocação de *stent* no canal arterial e cerclagem dos ramos da artéria pulmonar.

As imagens foram obtidas através de tomógrafo GE *Revolution* com 512 detectores e modulação eletrocardiográfica, posteriormente tratadas no *software Slic3r* para segmentação estrutural. Para a confecção dos protótipos, foi utilizado um filamento termoplástico de ácido poliático com 0,2mm de espessura e apoios em PVA no equipamento de impressão *ZMorph* VX/E. Após a impressão, os P3D foram comparados visualmente pelos autores com as imagens de ATC.

## Resultados

**Figura 1:**
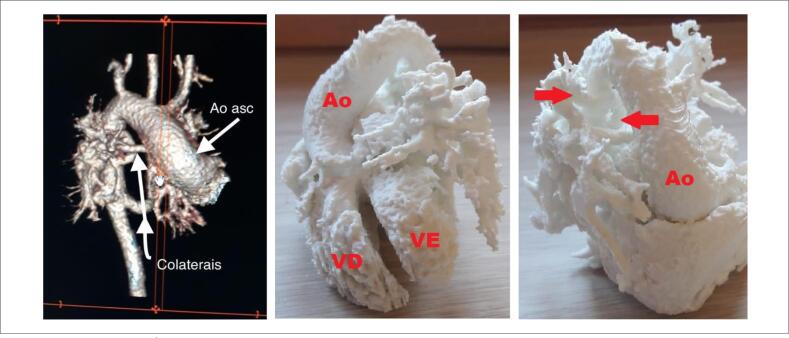
*Caso APu+CIV. À esquerda: ATC - setas indicam a aorta e a presença de artérias colaterais sistêmico pulmonares emergindo da aorta ascendente. À direita - P3D: visão frontal e lateral direita; Ao: aorta; VE: ventrículo esquerdo; VD: ventrículo direito*.

**Figura 2:**
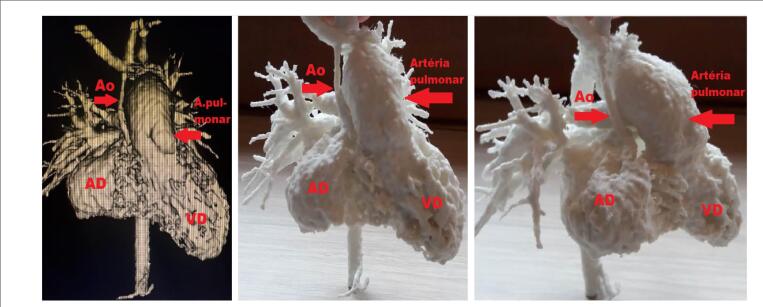
*Caso SHCE. À esquerda: ATC - setas indicam a aorta e a artéria pulmonar. À direita - P3D: visão lateral; Ao: aorta; AD: átrio direito; VD: ventrículo direito*.

Ao comparar visualmente as imagens da ATC com a impressão 3D, foi verificada a compatibilidade anatômica entre elas. Importante ressaltar que a percepção de detalhes importantes da anatomia no P3D foi evidenciada sob diversas perspectivas. No caso de APu+CIV, a compreensão da relação espacial entre as artérias colaterais sistêmico pulmonares originadas da aorta e os ramos pulmonares foi facilitada, bem como a observação comparativa das cavidades ventriculares (Figura 1). No caso de SHCE, foi visto o grau de hipoplasia da aorta ascendente e a presença de estenose do ramo pulmonar, além das relações espaciais entre elas (Figura 2). O tamanho dos modelos reconstituídos correspondeu à anatomia dos pacientes, possibilitando o estudo comparativo das dimensões entre as estruturas, o que pode colaborar na estratégia de tratamento cirúrgico. A possibilidade de ter o P3D em mãos e observar a anatomia sob diferentes ângulos tornou a compreensão das cardiopatias fácil e esclarecedora.

## Discussão

A impressão 3D é uma tecnologia que busca a complementação dos exames convencionais, uma vez que torna possível compreender melhor a malformação cardíaca. Ela proporciona o estudo detalhado sobre a localização, o comprimento, a extensão e a relação entre as estruturas malformadas, o que auxilia no planejamento cirúrgico e na identificação de detalhes anatômicos de pacientes submetidos a intervenções prévias.^6^ Por conseguinte, beneficia profissionais na ampliação do conhecimento da cardiopatia e proporciona um maior grau de segurança na escolha de técnicas cirúrgicas.^7^ Estudos em pacientes com atresia pulmonar e defeito do septo ventricular mostraram que P3D possibilitou a visualização de 96% da maioria das artérias colaterais aortopulmonares, se comparada ao intraoperatório. Dessa forma, focaliza a intervenção por cateterismo e reduz o tempo de procedimento, de exposição à anestesia, de fluidoterapia e do uso de contraste em procedimentos hemodinâmicos.^6^^–^^8^

A tecnologia P3D também pode ser utilizada em simulações cirúrgicas, visando a detectar a necessidade de adaptações no planejamento cirúrgico, reduzir complicações, obter bons resultados nos pós-procedimentos e promover o treinamento de estudantes e médicos.^1^ Alguns protótipos apresentam alta flexibilidade e não exigem manuseio especial, podendo ser aplicados antes e durante a intervenção cirúrgica.^9^

Na educação médica, ela possibilita ao graduando e ao médico-residente um maior entendimento da patologia e orientação espacial das estruturas. Com as dificuldades crescentes na obtenção de cadáveres para estudo, o uso de P3D é uma importante opção no ensino sobre a anatomia humana em escolas médicas.^10^

No contexto da relação entre médico e paciente, a possibilidade de os pais segurarem o protótipo em suas próprias mãos e visualizar os detalhes anatômicos descritos pelo profissional de saúde proporciona um melhor entendimento tanto da fisiopatologia relacionada quanto dos sintomas observados no paciente quanto ao tratamento. É descrito na literatura médica que os P3D auxiliam no estreitamento desta relação e são úteis na compreensão das informações e aumentam o conhecimento e o engajamento de paciente e familiares no que diz respeito à doença.^11^

A impressão em P3D é uma tecnologia em construção. Ela ainda apresenta limitações e desafios para garantir melhor qualidade ao produto. Entre elas, podemos destacar a precisão da montagem, a construção de modelos com mesmas propriedades mecânicas dos tecidos, o menor tempo de preparação e o custo econômico. A possibilidade de imprimir em cores facilitaria a identificação de diferentes tipos de estruturas como ventrículos, artéria pulmonar, ramos e aorta, principalmente no tocante ao ensino de graduação.

As cardiopatias representadas pelos protótipos satisfizeram as representações tridimensionais fidedignas às imagens obtidas nos exames de ATC, que foram utilizados como base para o estudo. A realização do P3D cardíaco é viável em nosso meio e pode ser uma ferramenta útil. Ela é capaz de auxiliar o clínico e a equipe cirúrgica na decisão terapêutica, o aprendizado do aluno no ensino médico de graduação, na especialização e na pós-graduação, o treinamento de habilidades cirúrgicas e o esclarecimento aos familiares da criança sobre a cardiopatia em tratamento.

Comentário: na publicação deste texto, devemos levar em consideração que as imagens dos P3D perdem muito de seu impacto visual se comparadas com a observação direta e real da peça em mãos.
