# Supplementary material for: Impressão de Protótipo Tridimensional (P3d) de Coração para Aplicação na Cardiologia Pediátrica: Uma Experiência Inicial
Source: Arq Bras Cardiol. 2021 Mar 3;116(3):507–9. [Article in Portuguese] doi: 10.36660/abc.20200086 (PMC8159552; doi:10.36660/abc.20200086)
Supplement: Translation

# Translation: Three-Dimensional-Printed Heart Prototype for Application in Pediatric Cardiology: An Initial Experiment

**Authors:** Maíra Levorato Basso, Alessandra Möbius Gebran, Julia Dullius Oliveira, Katrin Möbius Gebran, Letícia Carlota Bonatto, Maria Cecília Knoll Farah

**Affiliations:** 1 CuritibaPR Brazil Hospital Pequeno Príncipe, Curitiba, PR - Brazil; 2 CuritibaPR Brazil Faculdades Pequeno Principe, Curitiba, PR - Brazil

**Keywords:** Heart Defects Congenital, Coronary Angiography/methods, TomographyX-Ray Computed/methods, PrintingThree-Dimensional/trends

## Introduction

Three-dimensional (3D) prototyping technology is developing rapidly in medicine, and recent studies have shown its important applicability in pediatric cardiology.

Clinicians and surgeons use two-dimensional technologies, such as echocardiography, computed tomography (CT) angiography, and magnetic resonance imaging, to characterize structures and understand complex diseases. Because these images are often projected and extended on a flat screen, they may not represent the actual size of the structures, depth perception, or the proximity between them.^1^

The prevalence of congenital heart disease is estimated at 9.1 per 1000 live births.^2^ It causes significant hemodynamic and functional consequences and accounts for 6% of all infant deaths in the first year of life in Brazil.^3^ The diversity and complexity of heart diseases require surgeons to have a detailed understanding of anatomy for proper treatment planning and management and for providing a didactic explanation of the disease to family members.^4^^,^^5^

This study produced 3D-printed prototypes of congenital heart diseases from CT angiography image files as an initial experiment to increase scientific evidence.

## Methods

We conducted a descriptive observational study without the application of a numerical comparison tool. The study aimed to present our initial experience based on current evidence of the use of 3D technology and its benefits in understanding congenital heart diseases and their importance in our setting. Representative images of heart diseases were obtained from CT angiography image files and chosen among those of greater anatomical complexity in order to obtain better structural information with 3D printing. The selected heart diseases were pulmonary atresia with ventricular septal defect (PA-VSD) and systemic-to-pulmonary collaterals, and left ventricular hypoplasia (LVH) after ductus arteriosus stenting and cerclage of the branch pulmonary arteries. The images were obtained with a GE Revolution 512-slice CT scanner with electrocardiographic modulation and imported into Slic3r for structural segmentation. The prototypes were printed in a ZMorph VX/E 3D printer using 0.2-mm-thick polylactic acid thermoplastic filaments and PVA support. After printing, the 3D prototypes were visually compared with the CT angiography images by the authors.

## Results

Visual comparison of the CT angiography images with 3D-printed prototypes showed anatomical compatibility between them, and important anatomical details could be observed from different perspectives in the 3D prototypes. In the PA-VSD case, the 3D-printed prototype facilitated the understanding of the spatial relationship between the systemic-to-pulmonary collaterals originating from the aorta and the branch pulmonary arteries, as well as the comparative observation of the ventricular cavities (Figure 1).. In the LVH case, we could observe the degree of hypoplasia of the ascending aorta and the presence of branch pulmonary artery stenosis, in addition to the spatial relationships between them (Figure 2). The size of the 3D models corresponded to the anatomy of the patients, enabling a comparative study of the dimensions between the structures, which may contribute to the surgical treatment strategy. The possibility of having a hands-on 3D prototype to observe the anatomy from different angles allowed an easier and clearer understanding of heart diseases.

**Figure 1:** *PA-VSD case. Left: CT angiography: arrows indicate the aorta and the presence of systemic-to-pulmonary collateral arteries emerging from the ascending aorta. Right: 3D prototype – front and right-side view. Ao: aorta; LV: left ventricle; RV: right ventricle*.

**Figure 2:** *LVH case. Left: CT angiography – arrows indicate the aorta and pulmonary artery. Right: 3D prototype – lateral view. Ao: aorta; RA: right atrium; RV: right ventricle*.

## Discussion

Three-dimensional printing is a technology that aims to complement conventional tests, since it provides greater understanding of cardiac malformation. It allows a detailed study of the location, length, extension, and relationship between the malformed structures, thus assisting in surgical planning and in identifying anatomical details of patients who have undergone previous interventions.^6^ Consequently, it benefits surgeons by increasing their knowledge of the heart disease and by providing a greater degree of safety when choosing the surgical technique.^7^ Studies of patients with PA-VSD have shown that 3D prototypes enable the visualization of 96% of the major aortopulmonary collateral arteries compared with intraoperative assessment. Thus, it focuses on catheterization and reduces operative time, exposure to anesthesia, fluid therapy time, and the use of contrast agents in hemodynamic procedures.^6^^–^^8^

The 3D prototypes can also be used for surgical simulations, aiming to anticipate the need for adjustments to the surgical plan, to reduce complications, to obtain good postoperative results, and to train students and physicians.^1^ Some 3D prototypes are highly flexible and do not require special handling; therefore, they can be used both before and during surgery.^9^

In medical education, 3D printing allows medical students and residents to better understand the pathology and spatial orientation of the structures. With the growing difficulties in obtaining cadavers for study, the use of 3D prototypes is an important option for the teaching of human anatomy in medical schools.^10^

In the context of the doctor-patient relationship, the possibility that parents may hold the prototype in their own hands and visualize the anatomical details described by the physician provides a better understanding both of the pathophysiology related to the patient's symptoms and of the proposed treatment. The literature shows that 3D prototypes help to strengthen the doctor-patient relationship, are useful in understanding information, and increase the knowledge and engagement of patients and families in relation to the disease.^11^

Three-dimensional printing is a developing technology that has limitations and challenges to ensure a better-quality product, including assembly accuracy, construction of models with the same mechanical properties of the tissues, shorter preparation time, and lower economic cost. The possibility of printing different colors would facilitate the identification of different types of structures, such as ventricles, pulmonary artery and branches, and aorta, especially in undergraduate medical education.

The heart diseases represented by our 3D prototypes provided reliable 3D representations of the CT angiography images used as a basis for the study. Producing 3D-printed heart prototypes is feasible and can be a useful tool in our setting, as they can be used to assist clinicians and surgical teams in treatment decision-making, students’ learning in undergraduate and graduate medical education, and surgical-skill education and to provide explanations to family members about the child's heart disease.

Note: in the publication of this manuscript, it must be taken into account that the visual effect of the 3D prototypes is reduced on photographs, compared with the direct, actual handling of the product.
